# Niclosamide and its analogs are potent inhibitors of Wnt/β-catenin, mTOR and STAT3 signaling in ovarian cancer

**DOI:** 10.18632/oncotarget.13466

**Published:** 2016-11-19

**Authors:** Rebecca C. Arend, Angelina I. Londoño-Joshi, Abhishek Gangrade, Ashwini A. Katre, Chandrika Kurpad, Yonghe Li, Rajeev S. Samant, Pui-Kai Li, Charles N. Landen, Eddy S. Yang, Bertha Hidalgo, Ronald D. Alvarez, John Michael Straughn, Andres Forero, Donald J. Buchsbaum

**Affiliations:** ^1^ University of Alabama at Birmingham, Department of Obstetrics and Gynecology, Division of Gynecologic Oncology, Birmingham, AL, USA; ^2^ University of Alabama at Birmingham, Department of Radiation Oncology, Birmingham, AL, USA; ^3^ Southern Research Institute, Department of Oncology, Birmingham, AL, USA; ^4^ University of Alabama at Birmingham, Department of Pathology, Division of Molecular & Cellular Pathology, Birmingham, AL, USA; ^5^ Ohio State University, Department of Medicinal Chemistry and Pharmacognosy, Columbus, OH, USA; ^6^ University of Virginia, Department of Oncology, Division of Gynecologic Oncology, Charlottesville, VA, USA; ^7^ University of Alabama at Birmingham, Department of Epidemiology, Birmingham, AL, USA; ^8^ University of Alabama at Birmingham, Department of Medicine, Division of Hematology & Oncology, Birmingham, AL, USA

**Keywords:** ovarian cancer, chemoresistance, cancer stem cells, targeted therapy, Wnt pathway

## Abstract

Epithelial ovarian cancer (EOC) is the leading cause of gynecologic cancer mortality worldwide. Platinum-based therapy is the standard first line treatment and while most patients initially respond, resistance to chemotherapy usually arises. Major signaling pathways frequently upregulated in chemoresistant cells and important in the maintenance of cancer stem cells (CSCs) include Wnt/β-catenin, mTOR, and STAT3. The major objective of our study was to investigate the treatment of ovarian cancer with targeted agents that inhibit these three pathways. Here we demonstrate that niclosamide, a salicylamide derivative, and two synthetically manufactured niclosamide analogs (analog 11 and 32) caused significant inhibition of proliferation of two chemoresistant ovarian cancer cell lines (A2780cp20 and SKOV3Trip2), tumorspheres isolated from the ascites of EOC patients, and cells from a chemoresistant patient-derived xenograft (PDX). This work shows that all three agents significantly decreased the expression of proteins in the Wnt/β-catenin, mTOR and STAT3 pathways and preferentially targeted cells that expressed the ovarian CSC surface protein CD133. It also illustrates the potential of drug repurposing for chemoresistant EOC and can serve as a basis for pathway-oriented *in vivo* studies.

## INTRODUCTION

Epithelial ovarian cancer (EOC) was responsible for an estimated 14,000 deaths in the United States in 2015 making it the deadliest gynecologic malignancy [1]. Surgical resection in combination with platinum and taxane-based chemotherapy is the standard treatment for patients with EOC. Although the majority of patients with EOC become clinically disease-free after initial treatment, 75% of patients ultimately recur within 5 years. Developing more effective and durable treatments is a significant unmet medical need in this disease context. A subset of cells may survive first-line chemotherapy and ultimately be responsible for the development of clinically detectable recurrence. These cells may be cancer stem cells (CSCs) that are resistant to primary therapy and are responsible for the generation of progeny cancer cells [2].

Important pathways that are frequently upregulated in CSCs are the Wnt/β-catenin signaling pathway, the mammalian target of rapamycin (mTOR) pathway, and the signal transducer and activator of transcription-3 (STAT3) pathway [3, 4]. All three of these pathways have been shown to be associated with recurrence and development of chemoresistance in ovarian cancer [5, 6]. A well established *in vitro* model has been used to replicate the behavior of CSCs, which involves plating cells in serum-free media that contains growth factors in low-attachment plates so that they form spheres rather than adhere to the plate as single cells. Studies have shown that these tumorspheres have increased expression of ovarian CSC markers such as CD133 and ALDH1A1 [7]. When these cells are implanted into mice they are more tumorigenic than the bulk population of tumor cells. In order to successfully prevent ovarian cancer recurrence, it is imperative that therapies are developed to specifically target these chemoresistant CSCs. A therapeutic intervention that targets both the Wnt/β-catenin and mTOR/STAT3 pathways provides a promising approach towards the eradication of CSCs.

The traditional drug-development process takes a tremendous amount of time and is extremely costly with a very high failure rate. It has been estimated that it requires approximately a billion dollars and 10 years for a drug to be developed and placed on the market [8]. Drugs that have previously been used with known pharmacokinetics, pharmacodynamics and toxicity profiles provide an advantage over new drug discovery. New applications can be found for existing drugs, called drug repurposing, which can be valuable especially in diseases such as EOC. Niclosamide (trade name Niclocide) is a salicyclamide derivative in the antihelminth family which has been approved by the U.S. Food and Drug Administration for the treatment of tapeworms. This safe, inexpensive drug has been used in humans for nearly 50 years. Several investigators have independently performed quantitative high-throughput screening of > 4,000 clinically approved compounds and found niclosamide to be a potent anti-cancer compound [9, 10]. Screening of the NCI 60 human tumor cell line panel identified niclosamide as an inhibitor of the Wnt/β-catenin pathway [9]. Niclosamide is a potent mitochondrial uncoupler which can have an effect on cell-cycle arrest and apoptosis [9]. One criticism for pursuing the use of niclosamide as an anti-cancer drug is it poor water solubility (0.23 μg/mL) and poor systemic bioavailability (∼10%) [11]. Our group and others have made efforts to develop analogs of niclosamide. Analogs 11 and 32 were both synthesized by Dr. Pui-Kai Li at the Ohio State University in an attempt to improve the bioavailability and solubility and potentially enhance niclosamide's inhibition of proliferation. We previously described the structure and synthesis of these two compounds, which are shown in Supplementary Table S4 [12].

In this study, we investigated the effects of niclosamide, analog 11, and analog 32 on ovarian cancer cells and found that niclosamide and its analogs inhibited the Wnt/β-catenin, mTOR, and STAT3 pathways in chemoresistant ovarian cancer cell lines, in cells derived from a chemoresistant ovarian cancer patient-derived xenograft (PDX model), and tumorspheres cultured from cells isolated from the ascites of patients with ovarian cancer. Several other groups have also shown that niclosamide and niclosamide derivatives can inhibit mTOR and STAT3 signaling in various tumor types [13–19]. We previously showed that niclosamide and its analogs were anti-proliferative and targeted the Wnt pathway in >30 primary ovarian cancer patient ascites samples, some of which were clinically platinum resistant [12, 20]. In addition, we found that niclosamide specifically decreased the stem cell marker ALHD1A1 and the Wnt pathway surface receptor LRP6. In this current study we demonstrate that niclosamide not only targets the Wnt pathway, but it also targets the mTOR and the STAT3 pathways and specifically targets CD133+ CSCs and chemoresistant cells isolated from a PDX ovarian cancer model.

## RESULTS

### Anti-proliferation effects of niclosamide and its analogs, as single agents and in combination with chemotherapy

**Figure 1:**
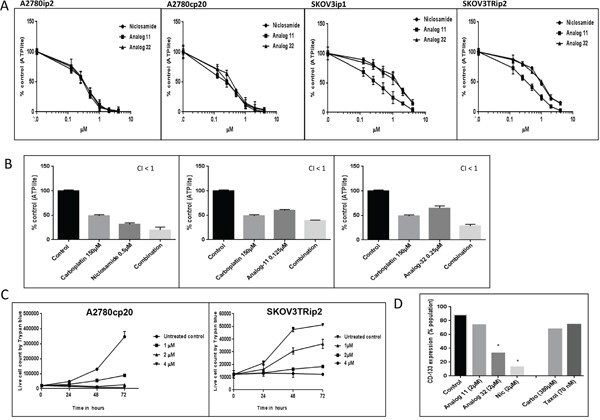
Anti-proliferative effects of niclosamide, and its analogs, as single agents and in combination with chemotherapy and expression of CD133 post treatment **A**. A2780ip2, A2780cp20, SKOV3ip1, SKOV3TRip2 cancer cell lines were treated with niclosamide, analog 11 or 32 (0.1- 4 μM) for 48 h. Level of ATP in the treated cells compared to the untreated cells were analyzed using ATPlite assay. **B**. A2780cp20 cells were treated concurrently with niclosamide or analogs in combination with carboplatin at indicated concentrations for 48 h. A combination index (CI) was calculated where CI <1 is synergistic. **C**. A2780cp20, SKOV3TRip2 cell lines were plated in 12 well plates and treated with niclosamide at indicated concentrations. Cell viability was measured by trypan blue exclusion method. All experiments were repeated 3 times. **D**. A2780cp20 cells were treated with niclosamide or analogs (2 μM), or IC_50_ doses of chemotherapy for 48 h and analyzed for CD133 expression by flow cytometry. Using student's t-test, *P < .05. All experiments were repeated 3 times. Data are represented as mean ± SD. Statistical analyses were performed using one-way ANOVA with application of Tukey's post test, P < .05 for all figures in (B).

Human ovarian cancer cell lines, A2780ip2 and SKOV3ip1 along with their platinum and taxane resistant derivatives A2780cp20 and SKOV3Trip2 were treated with niclosamide, analog 11, or analog 32 (0.1- 4 μM) for 48 h, and cell proliferation was assessed by measuring ATP levels using the ATPlite assay (Supplementary Figure S4). All three agents produced similar inhibition of proliferation, with IC_50_ values ranging from (0.41- 1.86 μM) (Figure 1A and Supplementary Table S1). These values are well below the known C_max_ of niclosamide in humans of 18.34 μM [21]. Analog 11 had slightly more anti-proliferative activity than the other two compounds for both the SKOV3ip1 parental cell line and SKOV3Trip2 taxane resistant line, but similar inhibition of proliferation was observed in the A2780ip2 and A2780cp20 cell lines. IC_50_ values for analog 32 were similar to niclosamide in all four cell lines.

We then investigated the effects of combining niclosamide, analog 11 or 32 with chemotherapy. The combination of niclosamide, analog 11, or analog 32 with carboplatin doses ranging from 50 -150 μM produced significantly greater inhibition of proliferation than either agent alone and statistical analysis indicated that the combination of the IC_50_ dose of carboplatin (150 μM) was synergistic with a combination index less than 1 (CI < 1) for all 3 agents in the A2780cp20 cell line (Figure 1B). Synergy was also observed with all 3 agents in the SKOV3ip1 and SKOV3Trip2 cell lines when combined with the IC_50_ dose of carboplatin, 100 μM and 50 μM respectively (Supplementary Figure S1). In the parental A2780ip2 cell line, niclosamide and analog 32 were synergistic with 50 μM of carboplatin, while analog 11 had an additive effect (CI = 1) (Supplementary Figure S1).

The anti-proliferative effects of niclosamide were validated in the two chemoresistant cell lines. Niclosamide inhibited proliferation in a time- and dose-dependent manner. When the difference in cell proliferation between treated and untreated cells was evaluated over a 72 h period in the two chemoresistant cell lines, 1 μM of niclosamide inhibited growth by 24 h and suspension of growth persisted for 72 h (Figure 1C and Supplementary Table S2). Niclosamide at 4 μM showed no increase in cell number after 72 h either due to cell kill, cell cycle arrest, or a combination of both.

In order to investigate if niclosamide and its analogs were targeting the CD133+ population of cells specifically, the chemoresistant A2780cp20 cells, which have a high expression of CD133+ compared to the parental line, were treated for 48 h with 2 μM of niclosamide, analog 11, or analog 32. At baseline, A2780cp20 cell line contains 80% CD133 + cells compared to 50-60% in the parental A2780ip2 cell line (Supplementary Figure S2B). Analog 32 and niclosamide both caused a statistically significant decrease in the CD133+ population with a 60-80% reduction of CD133+ cells (Figure 1D). There was a slight decrease in the CD133+ population after treatment with analog 11, but this was not statistically significant. Fifty percent of the total cells are killed with an IC_50_ dose of either carboplatin or paclitaxel, but >50% of the surviving cells still express CD133+; in contrast, niclosamide preferentially kills this population (Figure 1D).

### Inhibition of the Wnt/β-catenin pathway in ovarian cancer cell lines

**Figure 2:**
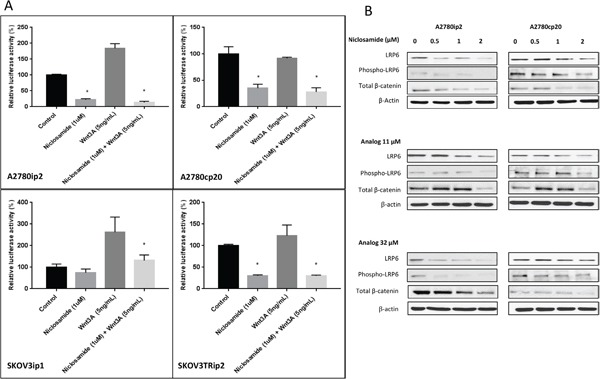
Wnt/β-Catenin specific inhibition of ovarian cancer cell lines **A**. A2780ip2, A2780cp20, SKOV3ip1 and SKOV3TRip2 cells in 24 well plates were treated with niclosamide and or Wnt3A, along with TOPflash construct and β-galactosidase-expressing vector in each well. After being incubated for 24 h, cells were analyzed for Wnt signaling. **B**. A2780ip2 and A2780cp20 cells were treated in 6 well plates with niclosamide and analogs at indicated concentrations for 24 h. The levels of LRP6 and phospo-LRP6, total β-catenin, were examined by western blot. All experiments were repeated 3 times. Data are represented as mean ± SD. Statistical analyses were performed by using student's t-tests, * P <0.05 when niclosamide group was compared to untreated control and niclosamide with Wnt3A group was compared to Wnt3A alone.

In order to evaluate niclosamide's ability to inhibit the Wnt/β-catenin pathway, we measured the nuclear β-catenin driven transcription activation after treatment with 1μM of niclosamide for 24 h using the TOPflash luciferase reporter assay. We observed a statistically significant suppression of β-catenin driven transcription activity in the presence of niclosamide in all four cell lines (Figure 2A). This suppression was also observed in both the parental A2780ip2 and SKOV3ip1 and the chemoresistant A2780cp20 and SKOV3Trip2 cell lines under Wnt stimulation (Figure 2A). Interestingly, we noted that in the chemoresistant lines A2780cp20 and SKOV3Trip2, the addition of Wnt3a failed to show increased activation of β-catenin driven signaling. One explanation for this could be that Wnt/β-catenin is already maximally upregulated in the chemoresistant cells, and the addition of Wnt3a does not further increase the level of nuclear β-catenin. It could also be possible that the Wnt/β-catenin pathway regulation of these cells is driven by other Wnt ligands and not Wnt3a or that it is deregulated downstream of Wnt stimulation due to other events that stabilize β-catenin.

LRP6 is an essential Wnt co-receptor for the Wnt/β-catenin signaling pathway, and LRP6 phosphorylation is critical for Wnt/β-catenin signaling activation induced by Wnt proteins. Uncomplexed cytosolic β-catenin (free β-catenin) can translocate to the cell nucleus and bind transcription factors leading to the transcription of Wnt target genes. To further evaluate niclosamide and its analogs' effect on the Wnt/β-catenin pathway, we used Western blot analysis to measure the transmembrane receptor LRP6, both total and phosphorylated, and the amount of total β-catenin. Niclosamide and its analogs inhibited endogenous LRP6 expression and phosphorylation in both parental A2780ip2 and platinum resistant A2780cp20 cell lines in a dose dependent manner (Figure 2B). We found that total β-catenin levels in both cell lines were significantly reduced after treatment with niclosamide, analog 11 and analog 32. Although analogs 11 and 32 decreased the expression of phosphorylated LRP6 and total β-catenin at the highest concentration in both cell lines, the decrease in nuclear β-catenin measured by TOPflash reporter assay was only statistically significant in the A2780cp20 cell line, but not in the parental A2780ip2 cell line (Supplementary Figure S2A). This could be the result of the analogs' ability to have an enhanced effect on the more stem-like cells. While analog 11 stimulated total β-catenin levels at 0.5 and 1μM using Western blot analysis in the A2780cp20 cell line, TOPflash reporter assay is a more specific measurement of Wnt/β-catenin pathway activation by measuring the nuclear β-catenin driven transcription activation, which showed a statistically significant decrease with 1μM of analog 11 (Supplementary Figure S2A).

### mTOR/STAT3 inhibition of ovarian cancer cell lines and patient samples

**Figure 3:**
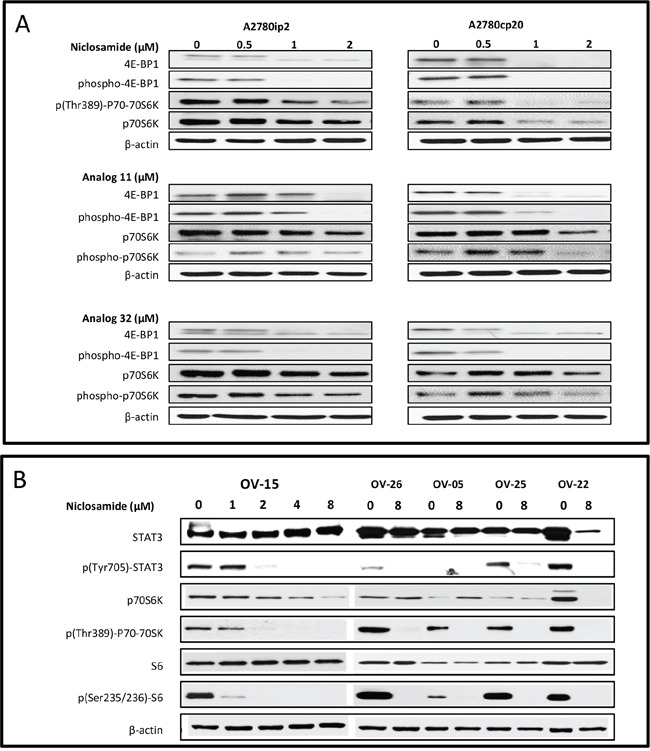
mTOR/STAT3 inhibition of ovarian cancer cell lines and patient samples **A**. A2780ip2, A2780cp20 cell lines were plated in 6 well plates and were treated with niclosamide and analogs 11 and 32 at indicated concentrations for 24 h. Treated lysates were examined for the levels of mTOR pathway proteins (4E-BP1, phospho-4E-BP1, p(Thr389)-P70-70S6K, p70S6K). **B**. Acites cells from ovarian cancer patients were plated in 6 well plates and were treated with niclosamide at indicated concentrations for 24 h. Treated lysates were examined for the levels of STAT3 pathway proteins (p(Tyr705)-STAT3 and STAT3) and mTOR pathway proteins (p(Thr389)-p70-70SK, p70S6K, p(Ser235/236)-S6, and S6).

Overexpression of downstream mTOR effectors 4E-BP1 and S6K lead to poor cancer prognosis [22]. Activation of mTOR leads to the phosphorylation of P70S6K at threonine 389. In order to evaluate the effect of niclosamide and its analogs on the mTOR pathway, we measured these proteins by Western blot analysis. As demonstrated in Figure 3A, 1 μM of niclosamide inhibited 4E-BP1 and phosphorylated 4E-BP1 in both parental A2780ip2 and chemoresistant A2780cp20 cell lines after 24 h. Analog 11 and analog 32 both inhibited mTOR pathway proteins 4E-BP1, phospho-4E-BP1, p70S6K; however, p(Thr389)-p70-70S6K was only consistently inhibited at the 2 μM dose (Figure 3A).

We have previously published that niclosamide is a potent STAT3 inhibitor in triple negative breast cancer cell lines [23]. To evaluate this finding in ovarian cancer patient samples, we measured total STAT3 and phosphorylated STAT3 after treatment with niclosamide. Niclosamide reduced STAT3 and mTOR downstream protein expression in ascites cells from ovarian cancer patients. STAT3 is phosphorylated by a kinase and translocates to the cell nucleus where it acts as a transcription activator. Specifically, STAT3 becomes activated after phosphorylation of tyrosine 705 in response to specific ligands. Western blot analysis of the phosphorylated (Tyr705) STAT3 protein after 24 h treatment with niclosamide (1 – 8 μM) showed a dose-response inhibition in ovarian cancer patient ascites sample OV-15. In OV-26, OV-05, OV-25 and OV-22, we used the highest dose in an effort to see the most significant effect and the 8 μM concentration of niclosamide completely abolished the phosphorylated (Tyr705) STAT3 protein expression (Figure 3B). Patient sample labeling (i.e. OV- patient #) was kept consistent with our previously published paper that showed dose-response inhibition of Wnt/β-catenin signaling proteins (LRP6, pLRP6, free and total β-catenin) and 3 target genes (Axin2, survivin, and cyclin D1) [20]. In addition, the mTOR pathway proteins p(Thr389)-P70-70SK and p(Ser235/236)S6 were significantly inhibited in the patient samples (Figure 3B). Overall, niclosamide was more consistent than analog 11 or analog 32 at inhibiting both STAT3 and mTOR protein expression.

### Cell cycle arrest and apoptosis by niclosamide

**Figure 4:**
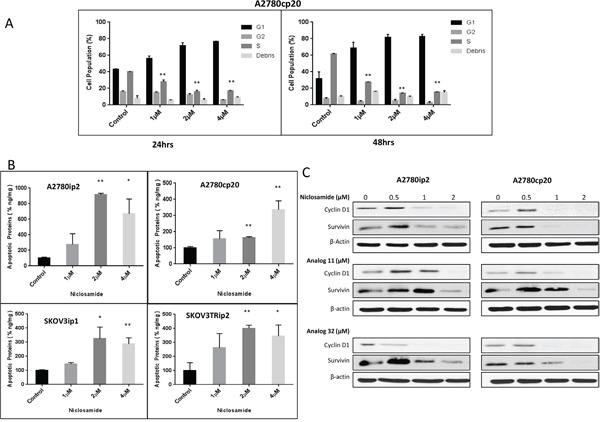
Cell cycle arrest by niclosamide and effect of niclosamide on cancer cell apoptosis and proliferation **A**. A2780cp20 cell line was plated in 12 well plates and treated with indicated concentrations of niclosamide. Cells were stained with PI as described in Materials and Methods. Percentages of cells in different phases of the cell cycle were determined by flow cytometry at 24 and 48 h. **B**. A2780ip2, A2780cp20, SKOV3ip1, SKOV3TRip2 cancer cell lines were treated with niclosamide at indicated concentrations for 48 h. Floating and attached cells were combined for apoptosis detection by Cell Death ELISA kit for histone–associated DNA fragments as described in Materials and Methods. **C**. A2780ip2, A2780cp20 cell lines were treated with indicated concentrations of niclosamide. Lysates from treated cell lines were analyzed for survivin and cyclin D1. Data are represented as mean ± SD. Statistical analyses were performed by using student's t-tests, *P<.05, **P<.005 for figures (A) and (B).

To elucidate the mechanism by which niclosamide inhibited cellular proliferation and caused cell death, we analyzed treated cells for both cell cycle arrest by flow cytometry and ELISA apoptosis assay. All cells were evaluated for their phase in the cell cycle by flow cytometry at 24 and 48 h after treatment with niclosamide. After treating A2780cp20 cells with niclosamide (1- 4 μM), cells arrested at G1 by 24 h at a 1 μM concentration, which was even more evident at higher concentrations (Figure 4A and Supplementary Figure S3).

Apoptosis was determined using an ELISA-apoptosis assay to detect mono- and oligonucleosomes released into the cytoplasm of apoptotic cells as a result of DNA degradation. After treatment with 2 μM of niclosamide, production of apoptotic DNA fragments was significantly increased compared to untreated controls in the A2780ip2, SKOV3ip1 and SKOV3Trip2 cell lines (Figure 4B). Four μM of niclosamide was required to see significant levels of apoptotic DNA fragments in the A2780cp20 chemoresistant cell line. This data suggests niclosamide is causing cell cycle arrest at lower concentrations and causing senescence and cell kill by apoptosis at higher concentrations. However, necrosis and autophagy, due to mTOR inhibition, could also account for cell kill [15].

Western blot analysis was performed to evaluate the effect of niclosamide and its analogs on the Wnt/β-catenin pathway target genes that affect cell cycle and apoptosis, namely cyclin D1 and survivin. At a concentration as low as 1μM of niclosamide, the proto-oncogene cyclin D1, which controls transition from G1 to S phase, is decreased in both A2780ip2 and A2780cp20 (Figure 4C). Protein levels of survivin, a protein that helps the cell to evade apoptosis, is dramatically reduced in both parental A2780ip2 and chemoresistant A2780cp20 cells at 2 μM (Figure 4C). A potential mechanism for cell cycle arrest and cell kill is the decrease in cyclin D1 and survivin. Similar results were obtained with analogs 11 and 32.

### Anti-proliferative effects of niclosamide and analogs 11 and 32 in a PDX model

**Figure 5:**
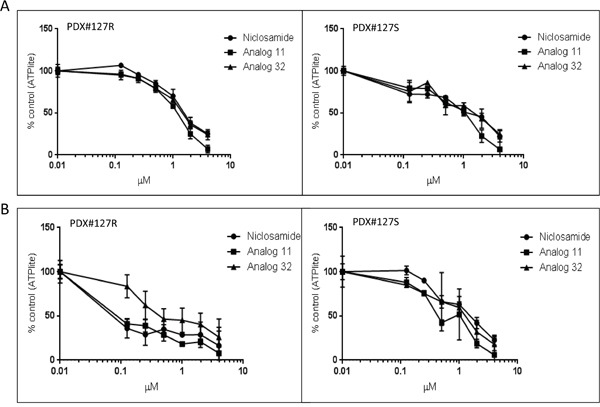
Anti-proliferative effect of niclosamide and analogs 11 and 32 on PDX mouse model cells PDX models 127 R (resistant) and 127 S (sensitive) were dissociated to single cell suspension and plated in 96 well plates. **A**. PDX cells were plated in tissue culture treated plates in 10% FBS + DMEM media. **B**. PDX cells were plated in low attachment plates with serum free x-vivo media with added supplements to promote stem cell growth. Cells were treated with niclosamide or analogs at increasing concentrations. Viability of cells was analyzed by ATPlite assay. All experiments were repeated 3 times. Data are represented as mean ± SD.

To confirm our cell line and previously published ascites tumorsphere *in vitro* observations, the effect of niclosamide treatment was evaluated in cells dissociated from an *in vivo* PDX model. A chemoresistant PDX model was created by serial chemotherapy treatments until the xenograft became resistant. Patient 127 had a histology of papillary serous adenocarcinoma and, as previously described in Dobbin *et al*, the histology of the tumor was maintained for 6 generations and a significant increase in the CSC markers ALDH1A1 and CD133 were seen in the PDX treated with carboplatin and paclitaxel for 4 weeks [24]. After dissociating cells from both the chemoresistant and the chemosensitive (parental) PDX model and plating cells both in tissue culture treated adherent conditions and in low attachment plates with stem-cell media, we treated all 4 types of cells with niclosamide, analog 11, or analog 32. In adherent conditions, the PDX#127-Resistant (127R) and PDX#127-Sensitive (127S) cells had similar sensitivity to all 3 agents (Figure 5A). When cells were grown in tumorsphere conditions, both niclosamide and analog 11 caused significantly more inhibition of proliferation (IC_50_ < 0.1) to the 127R chemoresistant cells compared to the 127S cells (IC_50_ < 1) (Figure 5B). This assay confirms our prior observations that niclosamide is able to preferentially target chemoresistant cells.

## DISCUSSION

Niclosamide, an FDA approved anthelmintic drug, is an inhibitor of Wnt co-receptor LRP6 and suppresses both the Wnt/β-catenin pathways and the mTOR/STAT3 pathway. Niclosamide has been shown to be anti-proliferative against prostate, colorectal, lung, breast, and ovarian cancers, and myelogenous leukemia by inhibiting multiple pathways (Wnt/β-catenin, Notch, NF-kB, mTOR, and STAT3) and inducing mitochondrial uncoupling [13, 16, 25–33]. In ovarian cancer, it has been shown to specifically kill CSCs [20, 23]. However, niclosamide exerts its antiparasitic activity in the intestinal lumen and has poor bioavailability, which limits its potential application as an anticancer agent. Niclosamide is a nitroaromatic coumpound, and the presence of a nitro group (NO_2_) in a compound can induce metabolic instability and serous toxicity. In the present study, we use two niclosamide analogs that we designed, synthesized, and biologically evaluated. In analog 11, we replaced the NO_2_ group in the anilide portion with another electron withdrawing group, trifluoromethyl (CF_3_). The CF_3_ group, which is widely used in current drug design, is metabolically more stable than the NO_2_ group. In analog 32, the salicyl portion was modified. Both analogs showed similar biological activity as niclosamide. They suppressed LRP6 expression, inhibited both Wnt/β-catenin and mTOR/STAT3 signaling in ovarian cancer ascites cells and chemoresistant cell lines, and displayed potent *in vitro* anti-proliferative effects. This data suggests that the novel niclosamide analogs are promising candidates for cancer therapy.

Interactions between the Wnt/β-catenin and STAT3/mTOR signaling pathways play an important role in ovarian carcinogenesis [34, 35]. There is significant cross-talk between these signaling pathways. The binding of a Wnt ligand to one of the transmembrane receptors LRP5/6 or Frizzled leads to the stabilization and translocation of active β-catenin in the cytoplasm. Stabilization and nuclear translocation of β-catenin leads to transcriptional activation of several important TCF/LEF target genes including survivin and cyclin D1 which are also regulated by mTOR signaling [36, 37]. Prior studies have shown that activated β-catenin causes enhanced STAT3 mRNA and protein expression [38]. In addition, studies have shown that activation of the STAT3 pathway leads to nuclear accumulation of β-catenin, causing increased levels of survivin and cyclin D1 [39]. Survivin is an oncogene that allows cells to recover from DNA damage and may contribute to chemotherapy resistance and help cells evade apoptosis. Cyclin D1 (*CCND1*), the proto-oncogene, is a cell-cycle regulator, which controls transition from G1 to S phase and has been shown to be correlated with platinum resistance. Given the high frequency of developing platinum resistance in ovarian cancer, it is imperative that we find treatments that target pathways that lead to chemotherapy resistance. Mechanistically, we found that niclosamide and two novel niclosamide analogs inhibit the production of both the oncogene survivin and the proto-oncogene cyclin D1, which both contribute to chemoresistance.

About 2% of ovarian cancer cells survive initial chemotherapy [40]. These CSCs have increased tumorgenicity, lead to recurrence, metastatic spread and chemoresistance. Wnt/β-catenin, mTOR, and STAT3 pathways have all been implicated in CSCs. ALDH1A1 and CD133 are two well established ovarian cancer stem cell markers. Both niclosamide and analog 32 preferentially killed the CD133+ population, which demonstrates that these agents could specifically target the CSCs. Niclosamide's ability to target the chemoresistant CSCs was also seen in the PDX model, where more effective inhibition of proliferation was seen in the cells dissociated from the chemoresistant PDX model and grown in stem cell media on low attachment plates.

Andrews *et al*. described a study where human volunteers received a single oral dose of 2,000 mg of carbonyl-14^c^-labeled niclosamide and the serum concentration was measured and found to be 0.25 - 6.0 μg/ml, which is equivalent to 0.7-18 μM [21]. These values are below the known C_max_ of niclosamide in humans of 18 μM. We show in the present study that niclosamide and analogs 11 and 32 inhibited ovarian cancer cell proliferation *in vitro* with IC_50_ values between 0.41 and 1.86 μM. Anti-proliferative effects were seen in established ovarian cancer cell lines, A2780ip2 and SKOV3ip1, as well as the chemoresistant A2780cp20 and SKOV3Trip2 cell lines, which are enriched for CSCs [41, 42]. Niclosamide and its analogs were able to reverse the resistance of A2780cp20 to carboplatin, suggesting that in women with platinum-resistant ovarian cancer these agents could reverse chemoresistance.

ATPlite assay was initially performed to evaluate the cytotoxicity of niclosamide and its analogs, but the ATP assay is an indirect measure of cell number based on constant ATP. The results could have been confounded by niclosamide's well known effect on oxidative phosphorylation in the mitochondria. Therefore, we used the trypan blue exclusion test in an effort to more accurately measure inhibition of proliferation. In order to better understand the anti-proliferative effect of the drugs, cell cycle arrest assay by flow cytometry and apoptosis ELISA assays were performed. Cell cycle arrest was seen at the G1 phase, which could be partially due to the decrease in the Wnt/β-catenin target gene, cyclin D1. While some amount of apoptosis was observed, this could be concentration dependent, contributing to only a small fraction of the biological response. The ATP level changes in the different cell types is likely a result of cell cycle arrest rather than cell kill.

Clinical trials are evaluating mTOR inhibitors, Wnt inhibitors, and STAT3 inhibitors in ovarian cancer and other solid tumors. Unfortunately, despite biological rationale and encouraging activity in preclinical models, trials of mTOR inhibitors in EOC have demonstrated disappointing results [43]. The Wnt/β-catenin and STAT3/mTOR pathways interact with each other and a number of other intracellular signaling networks, allowing for treatment escape when any single pathway inhibitor is used. Given the cross-talk between pathways and the adaptive capacity of cancer cells to evade targeted therapy, drug combinations are increasingly being investigated to abrogate both primary and acquired resistance to pathway specific targeted therapy. There has been evidence that aberrant expression of the Wnt pathway sensitizes cancer cells to an mTOR inhibitor; therefore, the use of a drug that targets both pathways would be even more effective than a single pathway inhibitor. Similarly, evasion from mTOR pathway inhibition could be due to activation and upregulation of the STAT3 pathway. The downside to multiple drug combinations is that these regimens can be toxic and difficult to simultaneously administer. A single agent which targets multiple pathways and has an existing safety profile, such as niclosamide is promising.

Drug repurposing is an emerging approach for identifying new indications for existing drugs. Niclosamide is an example where drug repurposing has uncovered an existing drug that targets molecular pathways involved in carcinogenesis. Niclosamide is well-tolerated in humans and an oral dose of 2,000 mg of niclosamide reaches blood concentrations in humans that caused significant cancer cell anti-proliferative activity in chemoresistant cells [21]. Additional *in vivo* studies need to be performed to further evaluate the bioavailability and anti-tumor effects of analog 11 and analog 32.

Currently, niclosamide is being evaluated in a clinical trial with enzalutamide in treating patients with androgen receptor positive, castration-resistant, metastatic prostate cancer (NCT02532114). In addition, there are two clinical trials registered on www.clinicaltrials.gov for the treatment of colon cancer by niclosamide (NCT02687009, NCT02519582) but neither are open yet for participant recruitment. Our findings suggest that niclosamide has antitumor activity for ovarian CSCs through the inhibition of multiple altered cellular pathways associated with metastasis and cancer recurrence. The observation that niclosamide can target the Wnt, mTOR and STAT3 pathways and reverse platinum resistance, provides compelling evidence to consider clinical studies with niclosamide in patients with ovarian cancer. In conclusion, niclosamide has a novel mechanism as an antineoplastic agent that should be evaluated in ovarian cancer patients.

## MATERIALS AND METHODS

### Reagents and cell culture

Niclosamide was purchased from Sigma-Aldrich (St. Louis, MO) and dissolved in DMSO to create a 4.8 mM stock solution, which was stored at 4°C. Analogs 11 and 32 were synthesized in the laboratory of Dr. Pui-Kai Li at The Ohio State University (Columbus, OH), dissolved in DMSO and stored at concentrations of 10 mM at -20°C. A schematic is shown in Figure 1 of the Haygood *et al*. manuscript [12]. Carboplatin was purchased from Sigma-Aldrich (St. Louis, MO), dissolved in water at concentration of 25 mM, and stored at 4°C. Paclitaxel was purchased from UAB hospital pharmacy.

The ovarian cancer cell lines A2780ip2, A2780cp20, and SKOV3TRip2 were acquired courtesy of Dr. Charles N Landen. SKOV3ip1 was acquired from the American Type Culture Collection (Manasses, VA). The cell lines were maintained in RPMI-1640 medium supplemented with 10% fetal bovine serum (Atlanta Biologicals, Fowery Branch, GA). A2780cp20 (platinum- and taxane-resistant), SKOV3TRip2 (taxane-resistant), were generated by sequential exposure to increasing concentrations of chemotherapy [44]. SKOV3TRip2 was maintained with the addition of 150 ng/ml of paclitaxel. All cell lines were routinely screened for *Mycoplasma* species (GenProbe detection kit; Fisher, Itasca, IL) with experiments performed at 70–80% confluent cultures. Purity of cell lines was confirmed with STR genomic analysis, and only cells less than 20 passages from stocks were used in experiments.

### Specimen collection and processing

Under IRB approval at UAB, all patients who were suspected to have ovarian cancer and scheduled to undergo surgery were consented for this study. Ascites fluid was collected at the time of laparotomy, diagnostic laparoscopy, or paracentesis. Cells were isolated from the ascites fluid via centrifugation at an initial 1000 rpm × 5 minutes, with serial spins at lower speeds (900–500 rpm), and finally at 400 rpm to remove red blood cells. Cell pellets were collected and washed in PBS, plated in ultra-low-attachment T-75 cm^2^ flasks (Corning Costar, Corning, NY) and incubated in X-vivo media (Lonza, Walkersville, MD) supplemented with 5 μg/mL insulin, and 20 ng/mL epidermal growth factor (PeproTech, Rocky Hill, NJ) in 37°C atmosphere and 5% CO_2_. Cells were cultured for 24 – 48 h and tumorspheres were then collected and stored at 10% DMSO and FBS for future analysis. In all samples, a board-certified gynecologic pathologist confirmed the cells to be ovarian cancer cells. PDX models 127S and 127R were developed in the laboratory of Dr. Landen. The PDX tissue was implanted in SCID mice. Tumors were harvested after reaching 15 mm x15 mm and were dissociated using Miltenyi Biotec Inc.(San Diego, CA) tissue homogenizer and tissue dissociation kits according to manufacturer's mouse tumor dissociation protocol. Dissociated tumors cells were plated in 96 well plates and analyzed by inhibition of proliferation assays after niclosamide or analog treatments.

### Viability assays

Cells from ovarian cancer cell lines were were plated in 96 well plates, 2000 cells per well, and exposed to increasing concentrations of niclosamide, analog 11, or analog 32 alone or in combination with carboplatin in triplicate. The cells were lysed after 48 h and assessed for viability by using ATPlite luminescence-based assay (PerkinElmer, Waltham, MA) according to the manufacturer's protocol. For the trypan blue exclusion method, ovarian cancer cell lines were seeded in 6 well plates at 8000 cells / well and treated with increasing concentrations of niclosamide. The cells were harvested at 0 h, 24 h, 48 h and 72 h and were counted for live cells.

### Western blot analysis

Cells from ovarian cancer cell lines or tumorspheres from ascites cells were seeded 1 million per well in 6 well plates and treated with niclosamide, analog 11, and analog 32 at the concentrations of 0.5 μM, 1 μM, 2 μM. Following 24 h, the cells were lysed in RIPA buffer supplemented with protease and phosphatase inhibitors and PMSF. Protein concentrations were determined with the BCA Protein Assay Kit (Pierce). Immunoblot analysis was carried out by standard techniques previously described [12]. Equal quantities of protein were subjected to SDS–PAGE under reducing conditions. Following transfer to immobilon-P membrane, successive incubations with anti-phosphorylated-LRP6, anti-LRP6, anti-total and free β-catenin, anti-survivin, anti-cyclin D1,4E-BP1, phosphor 4E-BP1, STAT-3, phosphor STAT-3, 70S6K, phospho70S6K, S6, phosphor S6 or anti-actin, and horseradish peroxidase-conjugated secondary antibody were carried out for 60 –120 minutes at room temperature (Supplementary Table S3). The immunoreactive proteins were quantified using the ECL system (PerkinElmer). Films showing immunoreactive bands were scanned by Kodak Digital Science DC120 Zoom Digital Camera (Kodak, Rochester, NY).

### TOPflash luciferase reporter assay

Cells from ovarian cancer cell lines were seeded 40,000 per 50 μL in 96 well plates. Following 24 h, cells were transfected with 200 ng of TCF/LEF luciferase reporter (TOPFlash) (plasmid courtesy of Dr. Randall Moon, Upstate Biotechnology, Lake Placid, NY). Cells were transfected using Lipofectamine TM 2000 (Invitrogen, Carlsbad, CA) in Opti-MEM (Gibco/Invitrogen) per the manufacturer's instructions. After 6 h, cells were treated with 1 μM niclosamide, analog 11, or analog 32 and assayed for luciferase activity with or without Wnt3a ligand 24 h post-treatment. Luciferase activity was measured using a Turner 20/20 luminometer (Promega, Madison, WI) and was normalized to the total protein concentration as reported previously [23]. The luciferase activity was normalized to untreated control and represented as the mean ± SE for a minimum of 3 replicates. Recombinant Human Wnt-3a Protein (100 ng/ml) R&D Systems Inc. Minneapolis, MN (Catalog #5036-WN-010) was used to stimulate β-catenin driven Wnt signaling.

### Cell cycle analysis

For cell cycle analysis, cells were treated with vehicle, 1μM, 2 μM and 4 μM doses of niclosamide for 48 h, trypsinized, and fixed in 100% ethanol overnight. The dead floating cells were also collected and combined with live cells for staining. Cells were then centrifuged, washed in PBS, and re-suspended in PBS containing 0.1% Triton X-100 (v/v), 200 μg/mL DNase-free RNase A and 20 μg/mL propidium iodide (PI). PI fluorescence was assessed by flow cytometry and the percentage of cells in G0/G1, S and G2/M phases was calculated by the cell cycle analysis module for Flow Cytometry Analysis Software (FlowJo v.7.6.1, Ashland, OR).

### Apoptosis detection

Apoptosis was assessed with cell death detection ELISA kit purchased from Sigma Aldrich (St. Louis, MO). Cells were treated with vehicle, 1μM, 2 μM and 4μM of niclosamide for 48 h. The spent medium containing floating cells was saved and kept on ice. The adherent cells were collected by gentle trypsinization and were combined with the floaters for pelleting by centrifugation. After gentle lysis of the cells with the buffer provided with the detection kit, the cell lysate was used for the ELISA test. The results were normalized by the protein content obtained from parallel plates with the cells being lysed using the buffer as described above for Western blotting. Apoptosis was detected by presence of histone associated DNA fragments that can be found in cell cytoplasm several hours before plasma membrane breakdown.

### CD133 expression in ovarian cancer cell lines

A2780cp20 cells were harvested after 48 h of treatment with niclosamide, analog 11, or analog 32 at 2 μM, or paclitaxel 70 nM, Carboplatin 380 μM. Cells were stained with CD133 antibody for 30 min on ice and were analyzed for CD133 expression by flow cytometry.

### Statistical analysis

Statistical significance for TOPflash, cell cycle analysis, and ELISA were determined by Student's t-test and *P* < 0.05 was considered significant. To test differences in treatment effects, ANOVAs and Tukey tests were performed. In cases when comparisons had to be made for greater than two groups, ANOVAs were run. We followed with Tukey tests to compare the means of every treatment to the means of every other treatment to identify differences between any two means. Calculations were performed using GraphPad Prism 6 software (GraphPad Software, La Jolla, CA).

The IC_50_ (half maximum inhibitory concentration) was defined as the log_10_ of the niclosamide or analog concentration producing 50% reduction in ATP levels (in counts per second) compared with the untreated ascites or cells. Cell viability was measured using a ratio of ATP levels for treated tumorspheres or cell lines to untreated controls (percent control). The combination index (CI) for the dose–effect relationship of niclosamide or the analogs and carboplatin were calculated based on the multiple drug-effect equation of Chou–Talalay for calculation of synergy [45, 46]. CI = *D*_A_/IC_*x*,A_ + *D*_B_/IC_X,B_ where IC_X,A_ and IC_X,B_ are concentrations of drugs producing *X* % inhibition for each respective drug alone, and *D*_A_ and *D*_B_ are concentrations of each drug in the mixture that yield *X* % inhibition. The CI curve or modified isobologram is generated by plotting CI vs. *X*, ranging from 0 to 100%. Drug interactions are readily identified at any level of inhibition. The resulting CI theorem offers quantitative definition for additive effect (CI = 1), synergism (CI < 1), and antagonism (CI > 1). The quantitative diagnostic plot was generated with Calcusyn software version 2.0 (Biosoft, Ferguson, MO). All data represents an average of at least four replicates. Error bars represent mean ± SE as indicated in the figure legends.

## SUPPLEMENTARY FIGURES AND TABLES


