# Correction: Niclosamide and its analogs are potent inhibitors of Wnt/β-catenin, mTOR and STAT3 signaling in ovarian cancer

**DOI:** 10.18632/oncotarget.25151

**Published:** 2018-04-10

**Authors:** Rebecca C. Arend, Angelina I. Londoño-Joshi, Abhishek Gangrade, Ashwini A. Katre, Chandrika Kurpad, Yonghe Li, Rajeev S. Samant, Pui-Kai Li, Charles N. Landen, Eddy S. Yang, Bertha Hidalgo, Ronald D. Alvarez, John Michael Straughn, Andres Forero, Donald J. Buchsbaum

**Affiliations:** ^1^ University of Alabama at Birmingham, Department of Obstetrics and Gynecology, Division of Gynecologic Oncology, Birmingham, AL, USA; ^2^ University of Alabama at Birmingham, Department of Radiation Oncology, Birmingham, AL, USA; ^3^ Southern Research Institute, Department of Oncology, Birmingham, AL, USA; ^4^ University of Alabama at Birmingham, Department of Pathology, Division of Molecular & Cellular Pathology, Birmingham, AL, USA; ^5^ Ohio State University, Department of Medicinal Chemistry and Pharmacognosy, Columbus, OH, USA; ^6^ University of Virginia, Department of Oncology, Division of Gynecologic Oncology, Charlottesville, VA, USA; ^7^ University of Alabama at Birmingham, Department of Epidemiology, Birmingham, AL, USA; ^8^ University of Alabama at Birmingham, Department of Medicine, Division of Hematology & Oncology, Birmingham, AL, USA

**This article has been corrected:** The correct Funding information is given below:

**FUNDING**

NIH grant support in the Comprehensive Flow Cytometry Core (CFCC) (P30 AR48311), Dr. Londono is funded by a NIH Research Training Program in Basic and Translation Oncology T32 Grant (T32CA183926). Dr. Arend is supported by the Foundation for Women’s Cancer (WeRoc/OChO Ovarian Cancer Research Grant), Norma Livingston Foundation, AAOGF/ABOG Career Development Award, and ACS-IRG (IRG-60-001-53-IRG). Dr. Hidalgo is supported by the NHLBI Diversity Supplement (HHSN268201300001) and a grant from the Robert Wood Johnson Foundation, New Connections Program.

Original article: Oncotarget. 2016; 7:86803-86815. https://doi.org/10.18632/oncotarget.13466

