# Supplementary material for: Niclosamide and its analogs are potent inhibitors of Wnt/β-catenin, mTOR and STAT3 signaling in ovarian cancer
Source: Oncotarget. 2016 Nov 19;7(52):86803–15. doi: 10.18632/oncotarget.13466 (PMC5349955; doi:10.18632/oncotarget.13466)
Supplement: Supplementary file 1 [file oncotarget-07-86803-s001.pdf]

Niclosamide and its analogs are potent inhibitors of Wnt/β-catenin, mTOR and STAT3 signaling in ovarian cancer

SUPPLEMENTARY FIGURES AND TABLES

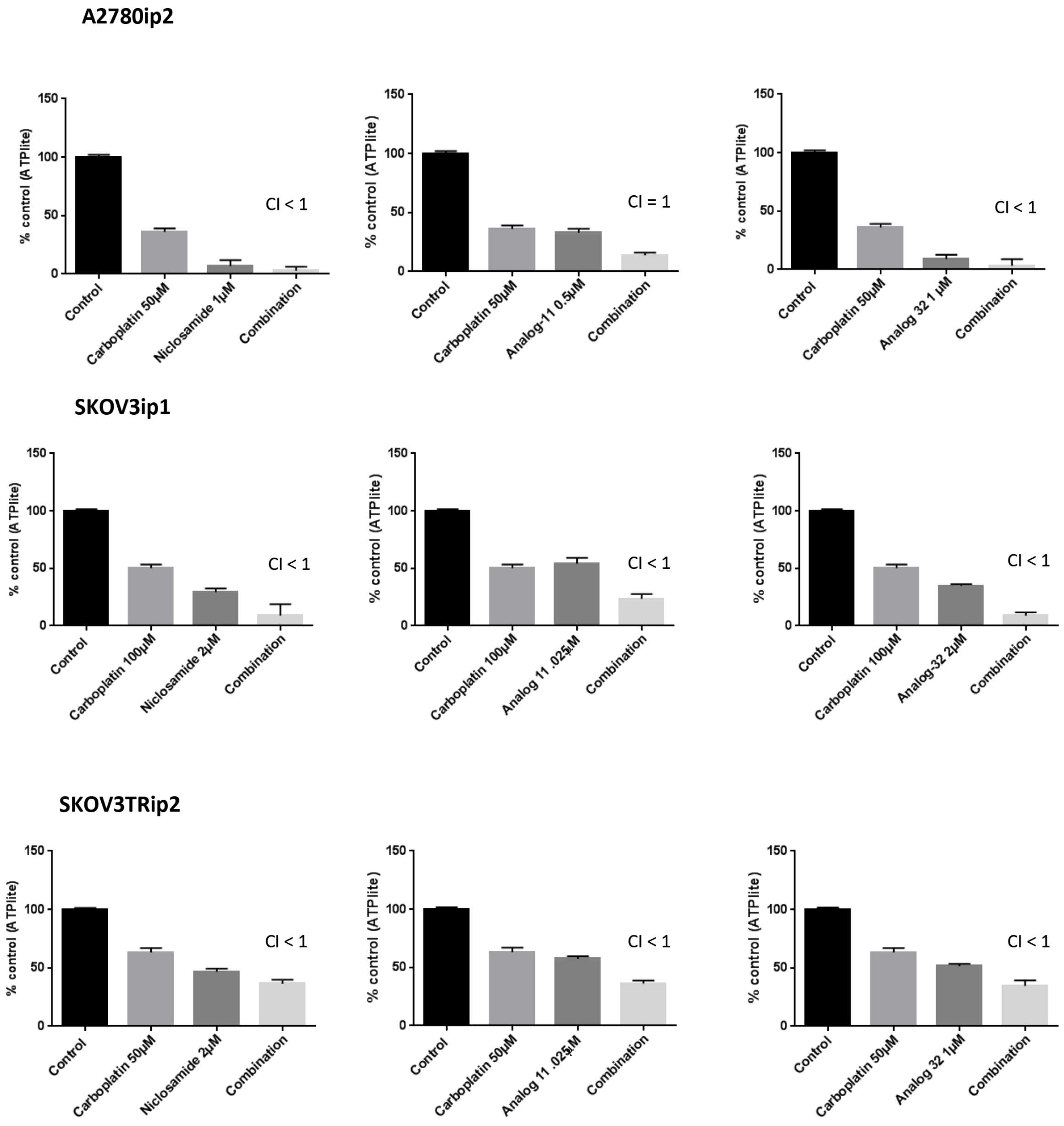

Supplementary Figure S1: Combination treatment of ovarian cancer cell lines. A2780ip2, SKOV3ip1 and SKOV3TRip2 cells were treated concurrently with niclosamide or analogs in combination with carboplatin at indicated concentrations for 48 h. All experiments were repeated 3 times. Data are represented as mean ± SD. Statistical analyses were performed by using one-way ANOVA with application of Tukey's post test, $P < .05$ when compared to untreated control for all figures. A combination index (CI) was calculated where CI <1 is synergistic and CI = 1 is additive.

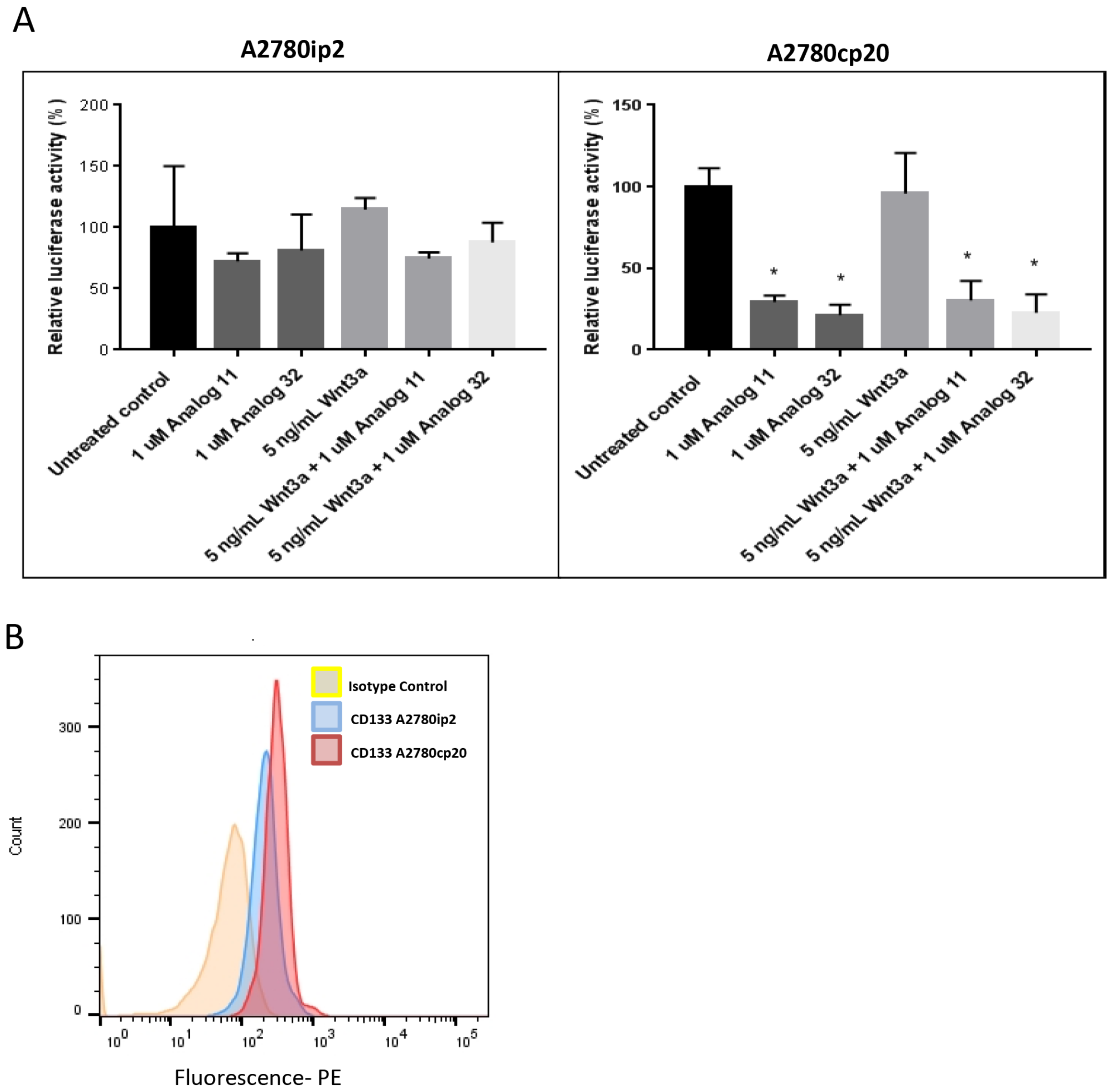

Supplementary Figure S2: TOPflash WNT activity and CD133 expression on A2780ip2 and A2780cp20 cells. A. A2780ip2 and A2780cp20 cells were treated in 24 well plates with 1 μM analog 11 and/or analog 32, Wnt3A and TOPflash construct and β-galactosidase-expressing vector in each well for 24 h and analyzed for WNT signaling. **B.** A2780ip2 and A280cp20 cells were stained for CD133 expression and compared to isotype control. Data are represented as mean ± SD. Statistical analyses were performed using student's t-test, *P < .05 when analog 11 or analog 32 group was compared to untreated control and analog 11 or analog 32 with Wnt3A group was compared to Wnt3A alone.

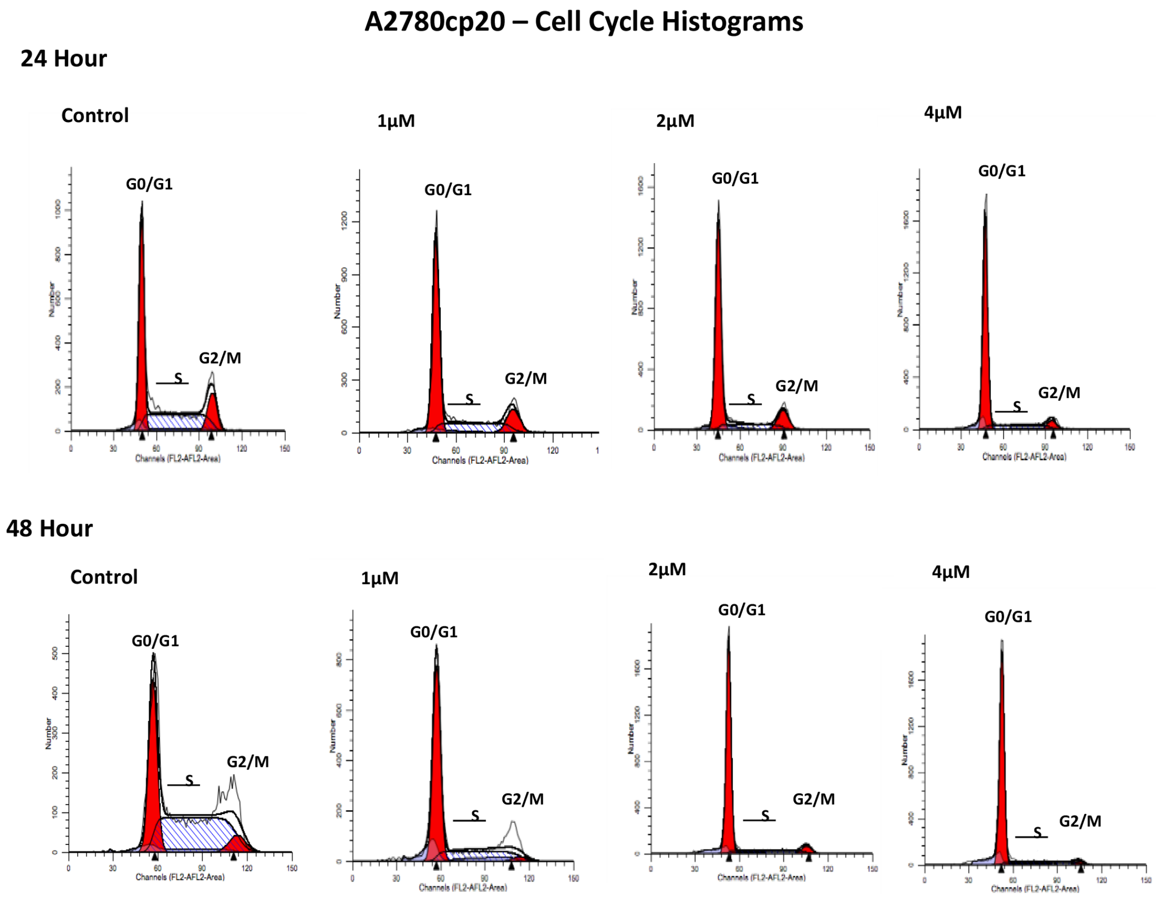

Supplementary Figure S3: Histograms of cell cycle arrest by niclosamide. A2780cp20 cell line was plated in 12 well plates and treated with indicated concentrations of niclosamide (1-4 µM). Cells were stained with PI as described in Materials and Methods. Percentages of population were determined by flow cytometry analysis at 24 and 48 h.

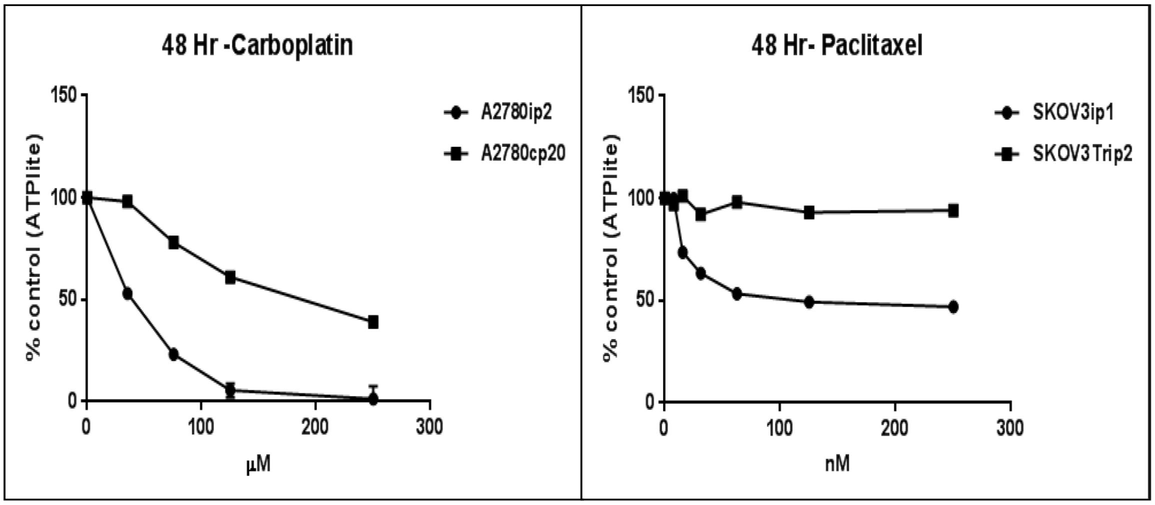

Supplementary Figure S4: Ovarian cancer cell lines resistance to carboplatin or paclitaxel. A2780ip2, A2780cp20 cells were treated with carboplatin (0 - 250 µM) for 48 h. SKOV3ip1 and SKOV3TRip2 cells were treated with paclitaxel at indicated concentrations (0 - 250 nM) for 48 h. Cells were analyzed for viability using ATPlite assay. All experiments were repeated 3 times. Data are represented as mean ± SD.

Supplementary Table S1: IC_{50} doses of niclosamide, analog 11 and analog 32 on ovarian cancer cell lines

IC_{50} µM	A2780ip2	A2780cp20	SKOV3ip1	SKOV3TRip2
**Niclosamide**	0.59	0.56	1.83	1.13
**Analog 11**	0.55	0.41	0.8	0.83
**Analog 32**	0.65	0.75	1.86	1.66

**A2780ip2, A2780cp20, SKOV3ip1, SKOV3TRip2 cancer cell lines were treated with niclosamide, analog 11 or 32 (0.1-4 µM) for 48 h. Cells were analyzed for viability using ATPlite assay. The niclosamide IC_{50} (half maximum inhibitory concentration) was defined as the log_{10} concentration generating a 50% reduction in ATP levels when compared with the untreated control.

Supplementary Table S2: Cell count by trypan blue exclusion method

SKOV3TRip2	Untreated control	1 µM	2 µM	4 µM
**Plating time**	8000	8000	8000	8000
**0 h**	12150	12150	12150	12150
**24 h**	21100	16175	13350	13050
**48 h**	47600	30600	16300	12800
**72 h**	51250	36250	18366	12166
**A2780cp20**	**Untreated control**	**1 µM**	**2 µM**	**4 µM**
**Plating time**	8000	8000	8000	8000
**0 h**	20000	20000	20000	20000
**24 h**	47291	28958	20416	15000
**48 h**	129250	53750	14000	8250
**72 h**	346050	88600	26133	7400

**A2780cp20, SKOV3TRip2 cell lines were plated in 12 well plates and treated with niclosamide at indicated concentrations. Cell viability was measured by trypan blue exclusion method.

Supplementary Table S3: Antibody information for western blotting

Number	Antibody	Concentration	Company Name and Catalog Number
1	Stat3	1:1000	Cell Signaling # 4904
2	P(Tyro705) stat3	1:500	Cell Signaling # 9131
3	P70S6K	1:1000	Cell Signaling # 9202
4	P(Thr389)P70-70SK	1:1000	Cell Signaling # 9205
5	S6	1:2000	Cell Signaling # 2217
6	P(Ser235/236)S6	1:2000	Cell Signaling # 4857
7	LRP6	1:1000	Cell Signaling # 3395
8	4E-BP1	1:1000	Cell Signaling # 9644
9	P4E-BP1	1:1000	Cell Signaling # 13443
10	Cyclin D1	1:1000	Cell Signaling # 2978
11	Survivin	1:1000	Cell Signaling # 2808

Supplementary Table S4: Structures of niclosamide and analog 11 and 32

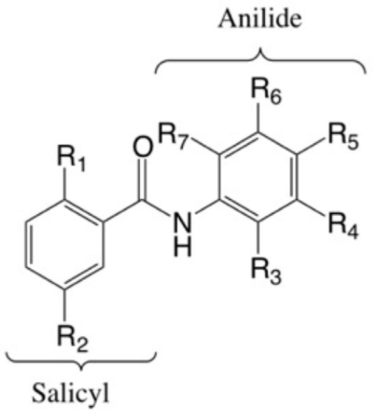

Compound	R¹	R²	R³	R⁴	R⁵	R⁶	R⁷
**Niclosamide**	OH	C1	C1	H	NO2	H	H
**Analog 11**	OH	C1	H	H	CF3	H	H
**Analog 32**	OONH2	C1	C1	H	NO2	H	H
